# Antitumor activity of a novel anti-vascular endothelial growth factor receptor-1 monoclonal antibody that does not interfere with ligand binding

**DOI:** 10.18632/oncotarget.12108

**Published:** 2016-09-19

**Authors:** Grazia Graziani, Federica Ruffini, Lucio Tentori, Manuel Scimeca, Annalisa S. Dorio, Maria Grazia Atzori, Cristina M. Failla, Veronica Morea, Elena Bonanno, Stefania D'Atri, Pedro M. Lacal

**Affiliations:** ^1^ Department of Systems Medicine, University of Rome Tor Vergata, Rome, Italy; ^2^ Laboratory of Molecular Oncology, “Istituto Dermopatico dell'Immacolata”-IRCCS, Rome, Italy; ^3^ Department of Biomedicine and Prevention, University of Rome Tor Vergata, Rome, Italy; ^4^ Laboratory of Experimental Immunology, “Istituto Dermopatico dell'Immacolata”-IRCCS, Rome, Italy; ^5^ National Research Council of Italy (CNR), Institute of Molecular Biology and Pathology, Rome, Italy

**Keywords:** VEGFR-1, PlGF, melanoma, angiogenesis, monocyte/macrophage

## Abstract

Vascular endothelial growth factor receptor-1 (VEGFR-1) is a tyrosine kinase transmembrane receptor that has also a soluble isoform containing most of the extracellular ligand binding domain (sVEGFR-1). VEGF-A binds to both VEGFR-2 and VEGFR-1, whereas placenta growth factor (PlGF) interacts exclusively with VEGFR-1. In this study we generated an anti-VEGFR-1 mAb (D16F7) by immunizing BALB/C mice with a peptide that we had previously reported to inhibit angiogenesis and endothelial cell migration induced by PlGF. D16F7 did not affect binding of VEGF-A or PlGF to VEGFR-1, thus allowing sVEGFR-1 to act as decoy receptor for these growth factors, but it hampered receptor homodimerization and activation. D16F7 inhibited both the chemotactic response of human endothelial, myelomonocytic and melanoma cells to VEGFR-1 ligands and vasculogenic mimicry by tumor cells. Moreover, D16F7 exerted *in vivo* antiangiogenic effects in a matrigel plug assay. Importantly, D16F7 inhibited tumor growth and was well tolerated by B6D2F1 mice injected with syngeneic B16F10 melanoma cells. The antitumor effect was associated with melanoma cell apoptosis, vascular abnormalities and decrease of both monocyte/macrophage infiltration and myeloid progenitor mobilization. For all the above, D16F7 may be exploited in the therapy of metastatic melanoma and other tumors or pathological conditions involving VEGFR-1 activation.

## INTRODUCTION

Vascular endothelial growth factor receptor-1 (VEGFR-1) is a tyrosine kinase receptor (TKR) that binds to VEGF family members VEGF-A, VEGF-B and placenta growth factor (PlGF) [1, 2]. VEGF-A also interacts with VEGFR-2, a TKR responsible for the activation of signal transduction pathways that mediate most VEGF-A biological effects [3].

VEGFR-1 is expressed in endothelial cells, during vessel formation and remodeling, macrophages and myoepithelial cells, favoring cell migration and survival [4–6]. Moreover, it is involved in the mobilization of myeloid bone marrow-derived cells that generate tumor-associated macrophages [1] and is frequently expressed in a variety of human cancers, of which it predicts poor prognosis and recurrence [1, 4]. In tumor cells, VEGFR-1 signaling inhibits apoptosis and induces chemoresistance [1, 7–9]. Conversely, VEGFR-1 neutralization by an antibody that blocks ligand binding prolongs survival of tumor bearing mice [10].

In addition to VEGFR-1 transmembrane form, cells produce a soluble receptor form (sVEGFR-1) that derives from alternative splicing of the same gene transcript [11] and comprises the first six Ig-like domains of membrane VEGFR-1 plus a specific 31 amino acid sequence at the C-terminal domain. Soluble VEGFR-1 includes the growth factor binding region of membrane VEGFR-1 (residues 1–656) and, thus, prevents VEGF-A and PlGF interaction with their transmembrane TKRs [12]. Moreover, it is a component of the extracellular matrix (ECM) and can directly interact with α5β1 integrin triggering cell adhesion and migration [13].

At variance with VEGF-A, PlGF exclusively binds to VEGFR-1. PlGF is a pleiotropic factor that promotes migration, proliferation and survival of endothelial cells and affects several cell types regulating various biological responses [14]. However, its role is redundant both during development and in health conditions. By contrast, PlGF plays an important disease-associated role because its expression, which is low or undetectable in most adult healthy tissues, is significantly up-regulated in a number of pathological conditions [1] and has a predominant role in the angiogenic and inflammatory switch that occurs in various diseases [14]. In particular, PlGF expression is up-regulated in different types of human cancers and is associated with a poor prognosis [15, 16]. PlGF promotes tumor cell invasion of the ECM and enhances the activity of selected matrix metalloproteinases [17]. Consistently, studies performed in murine models showed that a neutralizing antibody against PlGF is able to inhibit both growth and metastatic spreading of several tumors as well as to enhance the efficacy of chemotherapy without causing significant side-effects [1]. In transgenic mice that overexpress PlGF in the skin, intradermal injection of syngeneic melanoma cells resulted in stimulation of tumor growth, mobilization of endothelial and hematopoietic stem cells, increase in the number and size of melanoma-associated vessels and formation of lung metastases [17]. Moreover, we previously demonstrated that PlGF and VEGFR-1 are co-expressed in a large number of human melanoma cell lines [18] and, together with other groups, provided experimental evidence that the interaction of PlGF with VEGFR-1 might modulate cellular pathways important for melanoma cell proliferation, apoptosis and invasiveness [9, 17–20].

On this basis, VEGFR-1 blockade is expected to exert antitumor activity by three different mechanisms: a) inhibition of tumor-associated angiogenesis, by hampering endothelium activation in response to angiogenic factors released by tumor cells (i.e., VEGF-A and PlGF); b) reduction of hematopoietic precursors mobilization from the bone marrow and of tumor infiltration by myelomonocytic cells, which secrete cytokines and pro-angiogenic factors that may contribute to tumor aggressiveness and resistance to anti-VEGF-A therapies; c) direct effect on VEGFR-1 positive tumor cells, by inhibiting their invasiveness and survival.

Antiangiogenic therapies that have been used so far for the treatment of a variety of solid tumors hamper VEGF-A signaling mediated by both VEGFR-2 and VEGFR-1 or exclusively by VEGFR-2. In fact, the humanized monoclonal antibody (mAb) bevacizumab targets VEGF-A, thereby preventing activation of both VEGFRs; small molecules TK inhibitors (e.g., axitinib, cabozantinib, lenvatinib, pazopanib, regorafenib, sorafenib, sunitinib, vandetanib) interact with the catalytic domain of several TKRs, including VEGFRs; and the fully human mAb ramucirumab is directed against VEGFR-2 [21–23]. Unfortunately, therapeutic use of molecules that interfere with VEGF-A/VEGFR-2 signaling determines severe side effects (e.g., bleeding, wound healing delay, gastrointestinal perforations, hypertension, thromboembolic complications, proteinuria) due to physiological angiogenesis inhibition [24, 25]. On the other hand, molecules selectively targeting VEGFR-1 are expected to cause less toxic effects than molecules directed against VEGFR-2 or VEGF-A because i) PlGF is capable of transducing its own signals through phosphorylation of tyrosine residues distinct from those phosphorylated upon VEGFR-1 stimulation by VEGF-A [26]; and ii) VEGFR-1 does not play a relevant role in physiological angiogenesis in the adult.

Experimental approaches undertaken so far to selectively inhibit VEGFR-1 include targeted polymer-drug conjugates, VEGFR-1 antagonistic peptides or peptidomimetics and mAbs that block ligand binding to the receptor [4, 10, 27, 28]. In principle, however, these VEGFR-1 inhibitors may impair sVEGFR-1 antiangiogenic activity mediated by sequestering of VEGF-A and PlGF.

In this context, we have reported VEGFR-1 derived peptides (A4, and its shorter version B3) that efficiently inhibit endothelial cell migration induced by VEGFR-1 specific ligands without interfering with growth factor binding to the receptor [29]. Peptide B3 interaction with VEGFR-1 extracellular region interferes with receptor homodimerization and markedly hampers angiogenesis both *in vitro* (formation of tube-like structures in collagen gels) and *in vivo* (matrigel-plug assay in mice) [29]. However, peptides have some pharmacokinetics draw-backs (e.g., short half-life due to proteolytic cleavage) that may limit their use as potential drug candidates.

With the aim of exploring the therapeutic potential of VEGFR-1 blockade in melanoma with a metabolically stable molecule, we produced a mAb (i.e., D16F7) against peptide A4. D16F7 specifically counteracts VEGFR-1 activation and chemotactic response of endothelial, myelomonocytic and melanoma cells to VEGF-A and PlGF without altering ligand binding to the receptor. Therefore, D16F7 is predicted not to interfere with the physiological regulation of VEGF-A activity by sVEGFR-1. Remarkably, in a preclinical murine model D16F7 strongly reduces angiogenesis and melanoma growth.

## RESULTS

### Anti-VEGFR-1 D16F7 mAb inhibits human endothelial, melanoma and myelomonocytic cell migration *in vitro* and angiogenesis *in vivo*

D16F7 mAb specifically recognized VEGFR-1 in immunoblot analysis and did not cross-react with VEGFR-2 (Supplementary Figure S1A). Binding to VEGFR-1 was concentration-dependent, as demonstrated by the results of ELISA using 96-well plates coated with VEGFR-1/Fc chimera (Supplementary Figure S1B).

**Figure 1:**
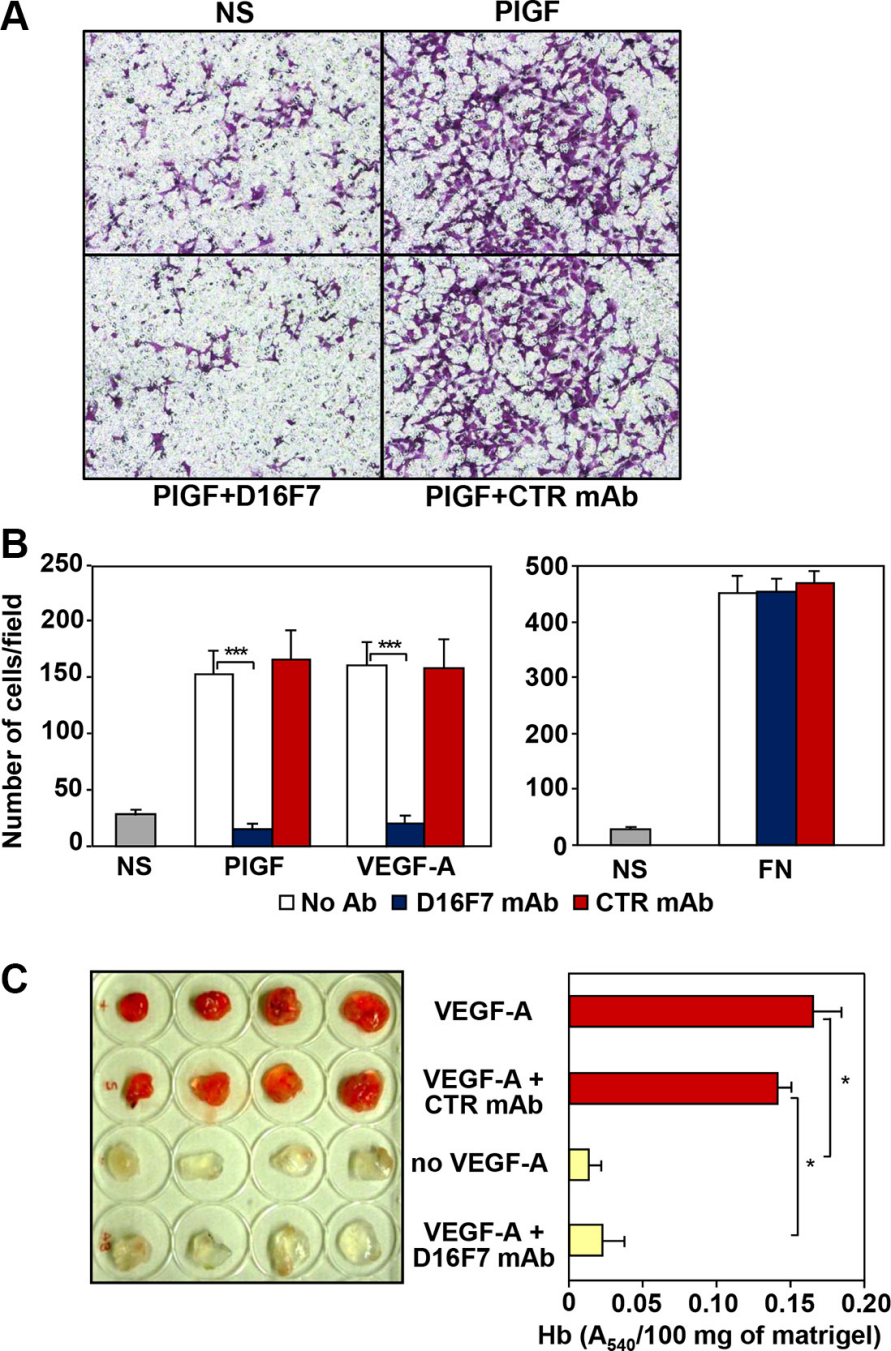
Effects of D16F7 mAb on endothelial cells (**A, B**) D16F7 mAb inhibits endothelial cells migration in response to PlGF and VEGF-A *in vitro*. Migration of HUV-ST cells (1.5 × 10^5^ cells/chamber, 18 h incubation), non stimulated (NS) or exposed to VEGFR-1 activating stimuli (50 ng/ml PlGF; 20 ng/ml VEGF-A) or to an unrelated stimulus (5 μg/ml fibronectin, FN), was tested in Boyden chambers containing gelatin coated filters in the presence or absence of 2.5 μg/ml of D16F7 mAb or control mouse IgG mAb (CTR mAb). Photographs from a representative experiment with PlGF out of three are shown (x200 magnification) (A). Histograms represent the arithmetic mean values of migrated cells/microscopic field ± SD of three independent determinations (B). (**C**) D16F7 mAb inhibits angiogenesis *in vivo*. Matrigel, containing (VEGF-A) or not (no VEGF-A) 100 ng/ml VEGF-A, was injected s.c. in the flank of C57BL/6 mice. In selected samples containing the angiogenic stimulus, 10 μg/ml D16F7 mAb or control mouse IgG mAb (CTR mAb) were included. The presence of newly formed blood vessels in matrigel plugs was observed macroscopically and the hemoglobin (Hb) content was quantified by the Drabkin's method. Histogram represents the mean values of the hemoglobin content, evaluated as A_540_/100 mg of matrigel in quadruplicate samples, ± SD.

D16F7 mAb ability to modulate the chemotactic response to PlGF and VEGF-A was investigated using human endothelial cells known to express VEGFR-1. Results indicated that D16F7 mAb strongly inhibited HUV-ST endothelial cell migration in response to VEGFR-1 ligands, whereas IgG control antibody did not have any effect (Figure 1A and 1B, left panel). D16F7 selectivity was demonstrated by the lack of activity on endothelial cell migration in response to an unrelated stimulus like fibronectin (Figure 1B, right panel).

D16F7 effects were also tested on murine endothelial cells using an *in vivo* matrigel plug assay. Angiogenesis was strongly induced five days after injection in C57BL/6 mice flank of matrigel plugs containing VEGF-A or VEGF-A plus control IgG as stimulus. By contrast, macroscopic analysis of the plugs that included VEGF-A plus D16F7 showed that newly formed blood vessels were not present, as in plugs where VEGF-A was not included (Figure 1C, left panel). Macroscopic analysis results were confirmed by quantitative measurement of hemoglobin content in the excised matrigel plugs (Figure 1C, right panel). These data demonstrate that D16F7 mAb possesses antiangiogenic activity and is able to cross-react with murine VEGFR-1. Indeed, the A4 peptide derived from human VEGFR-1, which had been used to produce D16F7 mAb, shares ~85% identity with the corresponding murine sequence (amino acids 149 to 161 in human and 150 to 162 in murine VEGFR-1).

**Figure 2:**
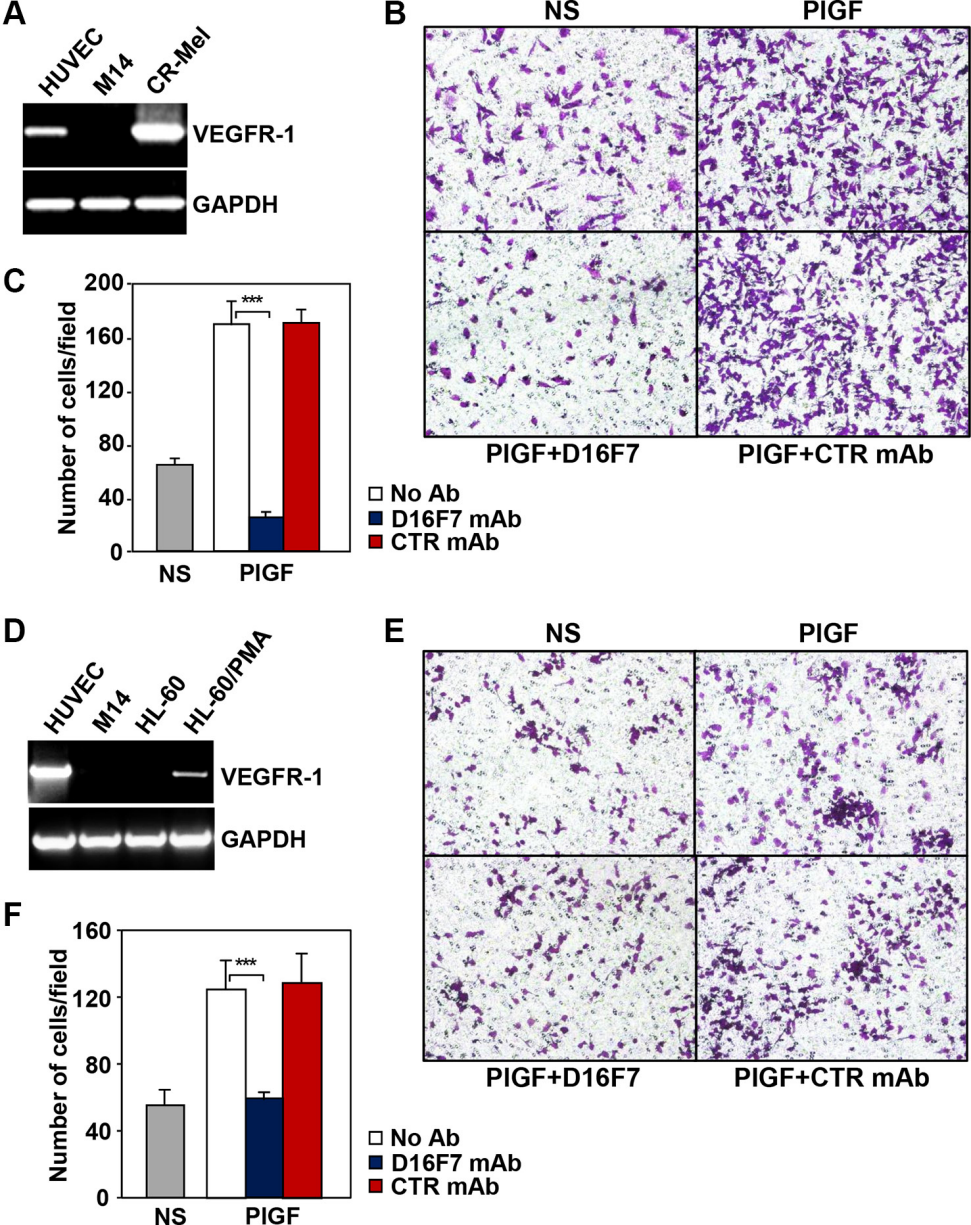
D16F7 mAb inhibits the migration of human melanoma and myelomonocytic cells that express VEGFR-1 in response to PlGF The CR-Mel melanoma cell line (**A, B, C**) and HL-60 promyelocytic cell line differentiated *in vitro* with PMA towards monocytic/macrophagic cells (**D, E, F**) were analyzed for VEGFR-1 expression (A, D) and the effect of D16F7 treatment on the chemotactic response to PlGF (B, C; E, F). VEGFR-1 expression was assessed by RT-PCR utilizing HUVEC and the melanoma cell line M14 as positive and negative controls, respectively. Migration of CR-Mel or differentiated HL-60 cells (2 × 10^5^ cells/chamber) in response to PlGF (50 ng/ml, 18 h incubation), was evaluated in Boyden chambers equipped with gelatin coated filters, in the presence or absence of 2.5 μg/ml D16F7 mAb or control mouse IgG mAb (CTR mAb). NS, non-stimulated cells. Photographs from a representative experiment out of three are shown (x200 magnification) (B, E). Histograms represent the arithmetic mean values of migrated cells/microscopic field ± SD of three independent determinations (C, F).

The down-modulating effect of D16F7 mAb on the migratory response of human melanoma cells to PlGF was analyzed using the CR-Mel cell line, which expresses VEGFR-1 (Figure 2A and [30]). Migration of CR-Mel cells exposed to PlGF was strongly down-modulated by D16F7, whereas it was not affected by control mAb (Figure 2B and 2C).

As a model of myelomonocytic cells, HL-60 cell line was induced to differentiate towards the monocytic/macrophage lineage by treatment with phorbol-miristate acetate (PMA). Differentiation of HL-60 cells by PMA was accompanied by VEGFR-1 expression induction (Figure 2D) and exposure to D16F7 mAb decreased cell migration triggered by PlGF to background values (Figure 2E and 2F).

Dose response experiments, aimed at calculating the D16F7 IC_50_ on PlGF induced cell migration, led to the following results: 0.48 ± 0.08 μg/ml for HUV-ST endothelial cells; 0.59 ± 0.17 μg/ml for CR-Mel; and 0.12 ± 0.02 μg/ml for myelomonocytic HL-60 cells.

### D16F7 inhibits VEGFR-1 phosphorylation without affecting ligand binding

**Figure 3:**
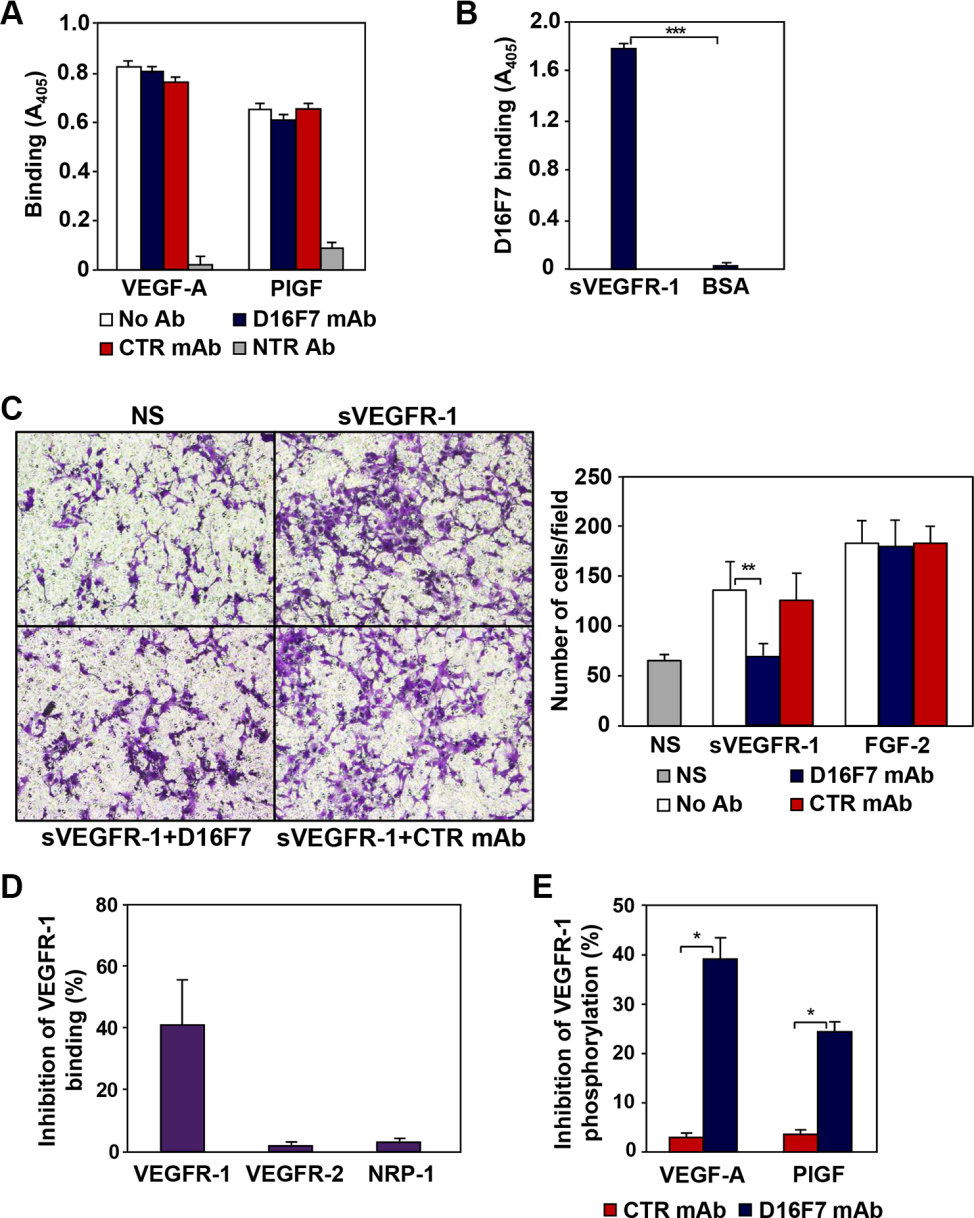
Mechanism of action of anti-VEGFR-1 D16F7 mAb (**A**) D16F7 binding to VEGFR-1 does not affect VEGFR-1 interaction with PlGF or VEGF-A. The influence of D16F7 mAb on PlGF and VEGF-A binding to VEGFR-1 was analyzed using 96-well plates precoated with VEGFR-1/Fc chimera. Selected wells were pre-incubated with 5 μg/ml D16F7 mAb or control mouse IgG mAb (CTR mAb). VEGF-A or PlGF were allowed to bind the receptor, alone (No Ab) or in combination with neutralizing antibodies (NTR Ab). VEGF-A or PlGF bound to VEGFR-1/Fc chimera were detected using biotinylated anti-VEGF-A or anti-PlGF mAbs and streptavidin-alkaline phosphatase-conjugated. (**B**) D16F7 binding to VEGFR-1. The mAb binding to VEGFR-1 was assessed by incubating selected VEGFR-1/Fc chimera or BSA precoated wells with the mAb and using an alkaline phosphatase-conjugated anti-mouse antibody. The results (A, B) are expressed as absorbance at 405 nm, and represent the arithmetic mean values ± SD of three independent determinations. (**C**) D16F7 mAb inhibits endothelial cell migration in response to sVEGFR-1. Migration of HUV-ST cells (1.5 × 10^5^ cells/chamber, 18 h incubation) induced by 5 μg/ml recombinant sVEGFR-1 or by 100 ng/ml FGF-2 was tested in Boyden chambers containing gelatin coated filters. Assays were performed by including 5 μg/ml D16F7 mAb or control mouse IgG mAb (CTR mAb) together with sVEGFR-1 or FGF-2 in the lower compartment of the chamber. Photographs from a representative experiment out of three are shown (×200 magnification) (left panel). Histograms represent the arithmetic mean values of migrated cells/microscopic field ± SD of three independent determinations. NS, non-stimulated cells (right panel). (**D**) D16F7 mAb hampers VEGFR-1 homodimerization. The influence of D16F7 mAb on VEGFR-1 homodimerization or heterodimerization with other VEGF-A receptors was evaluated in plates coated with sVEGFR-1. Chimeras containing the extracellular region of the different VEGFRs (i.e., VEGFR-1, VEGFR-2 and NRP-1) and the Fc region of human immunoglobulins were allowed to bind to the sVEGFR-1 on the wells previously treated or not with 5 μg/ml of D16F7 mAb. Results are expressed as percentage of binding inhibition and represent the mean values ± SD of six independent determinations. (**E**) D16F7 mAb inhibits VEGFR-1 auto-phosphorylation in response to VEGF-A and PlGF. VEGFR-1 auto-phosphorylation was analyzed by ELISA using extracts of CR-Mel cells stimulated at 37°C with VEGF-A or PlGF (200 ng/ml) for 10 min, after incubation of the cells with 5 μg/ml of D16F7 mAb or control mouse IgG mAb (CTR mAb) for 30 min. Results are expressed as percentage of binding inhibition and represent the mean values ± SD of three independent determinations.

To shed light on D16F7 mechanism of action, antibody effect on VEGFR-1 ligand binding and TKR activity was evaluated. Inspection of the three-dimensional structure of VEGFR-1 II IgG-like domain, involved in VEGF-A and PlGF binding [31, 32] showed that peptide A4, which had been used as immunogen to produce D16F7 mAb, does not overlap with VEGFR-1 regions involved in growth factor binding. Therefore, D16F7 was expected not to interfere with VEGFR-1 ligand binding ability. Indeed, in 96-well plates pre-coated with VEGFR-1/Fc and saturated with D16F7 mAb, either VEGF-A or PlGF ligands, which were used at saturating concentrations to obtain the highest signal, were able to bind VEGFR-1 at high levels (Figure 3A). Conversely, specific VEGF-A or PlGF neutralizing antibodies blocked growth factors binding to VEGFR-1 (Figure 3A, NTR Ab). The ability of VEGFR-1 extracellular region to strongly bind D16F7 mAb (Figure 3B) and, at the same time, VEGF-A or PlGF, demonstrates that mAb epitope and ligand binding sites are not overlapping, as expected. Since sVEGFR-1 comprises the entire growth factor binding region of the receptor, D16F7 mAb does not hamper sVEGFR-1 ability to bind VEGF-A or PlGF either. Therefore, in the presence of D16F7 mAb, the antiangiogenic activity of sVEGFR-1, which consists in the sequestration of VEGF-A and PlGF with consequent reduction of the amount of growth factors available to interact with membrane TKRs, is maintained, allowing sVEGFR-1 to continue to act as a decoy receptor.

Nevertheless, D16F7 incubation with sVEGFR-1 inhibited the chemotactic response of endothelial HUV-ST cells to the soluble receptor, but it did not affect chemotaxis induced by FGF-2 that was used as VEGFR-1 unrelated stimulus (Figure 3C), suggesting that mAb binding likely hinders sVEGFR-1 interaction with surface molecules involved in endothelial cell migration.

Even though ligand binding was not altered, D16F7 interaction with VEGFR-1 partially abrogated receptor homodimer formation (Figure 3D), which is required to trigger downstream signal transduction [33]. On the other hand, D16F7 did not affect VEGFR-1 interaction with VEGFR-2 or the co-receptor NRP-1 (Figure 3D). The mAb also inhibited VEGFR-1 autophosphorylation in response to its ligands, as shown in extracts of CR- Mel cells stimulated with either VEGF-A or PlGF (Figure 3E). Moreover, since the interaction of VEGFR-1 extracellular region with NRP-1 or integrins might affect cell adhesion, the effect of D16F7 on CR-Mel adhesion to different ECM components was tested. Cell pre-incubation with the mAb did not affect melanoma cell adhesion to matrigel, fibronectin, collagen I or gelatin and tumor cell proliferation (data not shown).

### D16F7 inhibits migration and vasculogenic mimicry of murine melanoma cells

**Figure 4:**
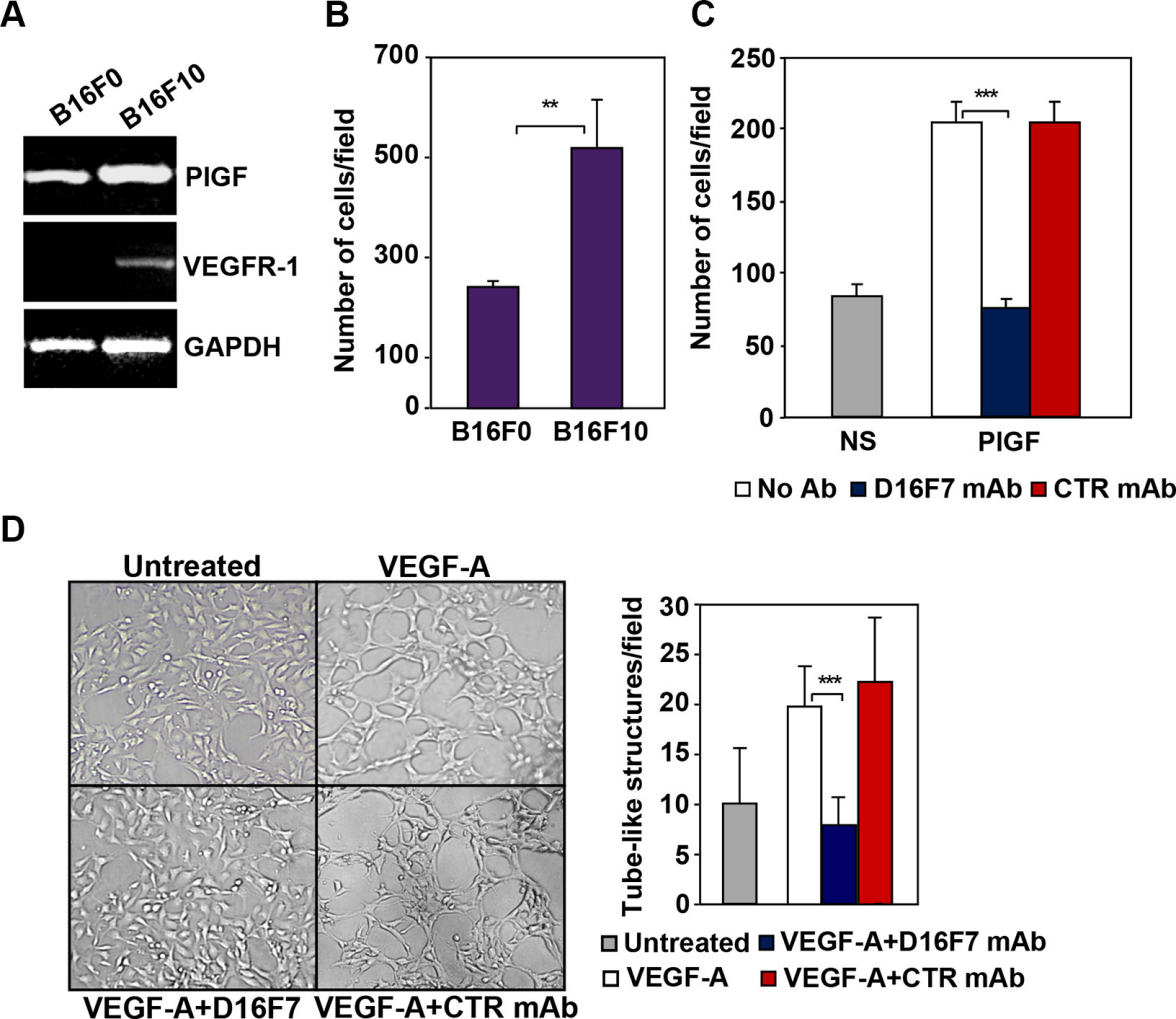
D16F7 inhibits migration and vasculogenic mimicry of murine melanoma cells (**A**) B16F10 cells but not B16F0 cells express VEGFR-1. PlGF and VEGFR-1 expression in B16F0 and B16F10 cells was evaluated by RT-PCR analysis. (**B**) B16F10 cells are characterized by a highly invasive phenotype. The ability of B16F0 and B16F10 cells to invade the extracellular matrix was analyzed in Boyden chambers equipped with matrigel coated filters (2 × 10^5^ cells/chamber, 4 h incubation). Histogram represents the arithmetic mean values of the number of invading cells/field ± SD of three independent determinations. (**C**) D16F7 mAb inhibits D16F10 cell migration in response to PlGF. Migration of B16F10 cells (1.5 × 10^5^ cells/chamber) induced by PlGF (50 ng/ml, 18 h incubation) was evaluated in Boyden chambers equipped with gelatin coated filters, in the absence (No Ab) or presence of 2.5 μg/ml D16F7 mAb or control mouse IgG mAb (CTR mAb). Histogram represents the mean values of the number of migrated cells/field ± SD of three independent determinations. NS, non-stimulated. (**D**) Effect of D16F7 mAb on B16F10 cell ability to form tube-like structures. Cells (4 × 10^4^/well), untreated or pre-incubated with 5 μg/ml D16F7 mAb or control mouse IgG mAb at room temperature for 30 min, were seeded in the absence or presence of 50 ng/ml VEGF-A on 24-wells plates coated with matrigel. Tube-like structures formation was analyzed after 48 h. Photographs from a representative experiment out of three are shown (×50 magnification). Histogram represents the arithmetic mean values of the number of tube-like structures ± SD, counted in ten different microscopic fields for each experimental group, for a representative experiment out of three.

The therapeutic potential of D16F7 was evaluated in a murine melanoma model. The relevance of VEGFR-1 in tumor aggressiveness in this model was initially characterized *in vitro*. In particular, two clones of B16 melanoma cell line were analyzed for PlGF and VEGFR-1 expression (Figure 4A) and ability to invade the ECM (Figure 4B) as index of tumor aggressiveness. Both B16F0 and B16F10 cell lines produced PlGF but only the more invasive B16F10 clone, expressed VEGFR-1 (Figure 4A). Further analysis demonstrated marked migration of B16F10 cells in response to PlGF that was strongly inhibited by D16F7 treatment (Figure 4C).

VEGF-A has been reported to enhance the ability of B16F10 cells to form tube-like structures similar to those produced by endothelial cells when cultured on matrigel *in vitro* (vasculogenic mimicry) [34]. Interestingly, D16F7 mAb significantly down-regulated the number of tube-like structures formed by melanoma cells upon exposure to VEGF-A, whereas control IgG did not have any significant effect (Figure 4D).

By contrast, D16F7 neither inhibited proliferation nor induced apoptosis of B16F10 melanoma cells *in vitro* (data not shown).

Therefore, VEGFR-1 positive B16F10 cells were selected for *in vivo* studies with D16F7.

### D16F7 inhibits *in vivo* melanoma growth, monocyte/macrophage infiltration and hematopoietic progenitor mobilization

**Figure 5:**
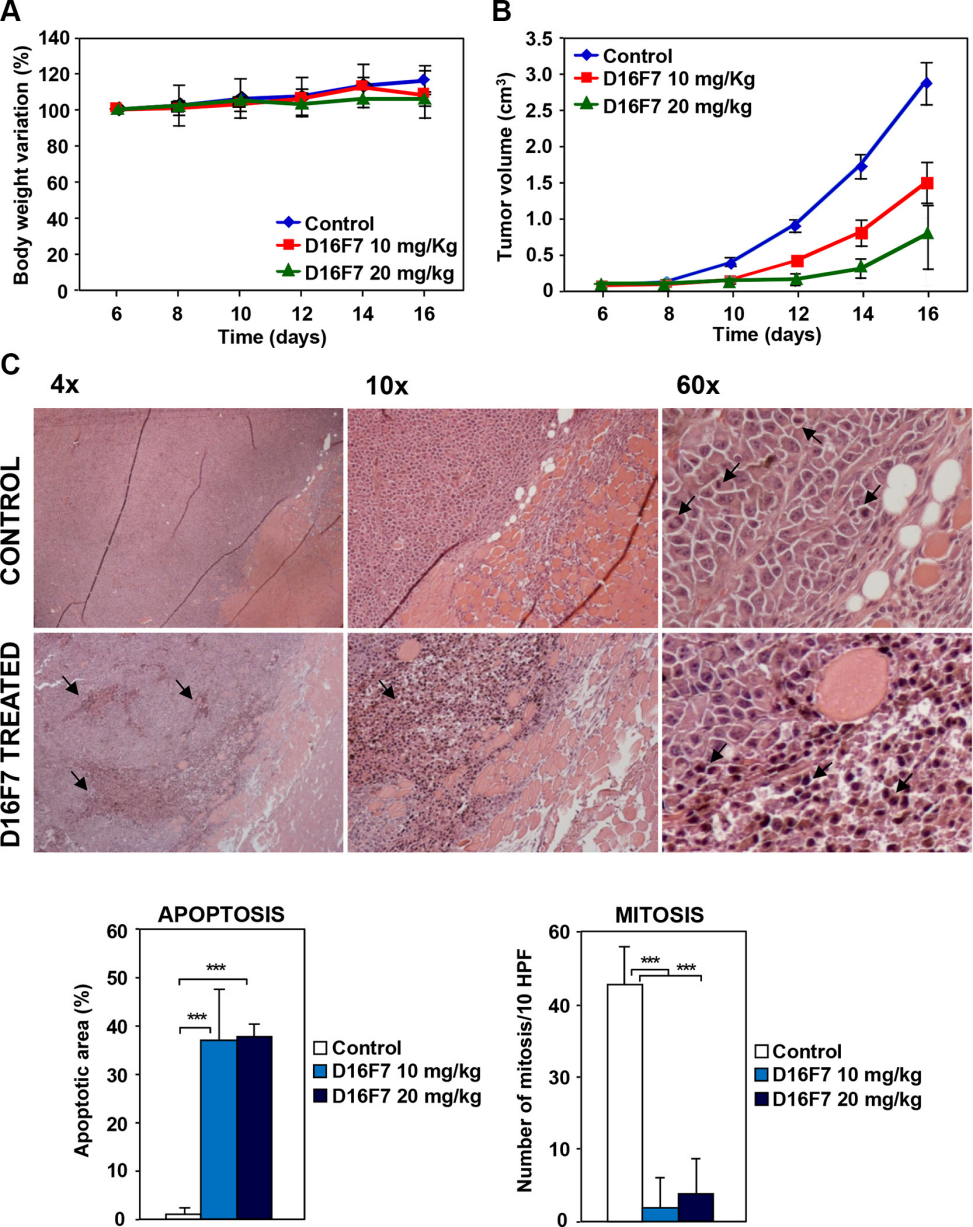
Effects of D16F7 mAb treatment on tumor cells in an *in vivo* murine model (**A**) Safety analysis of D16F7 treatment. Toxicity of D16F7 mAb treatment was evaluated on the basis of net body weight reduction in B6D2F1 mice treated with five doses of the indicated amounts of the antibody (8 animals/group), as compared to control animals. (**B**) D16F7 mAb inhibits *in vivo* tumor growth. B16F10 melanoma cells (2.5 × 10^5^/mouse) were injected i.m. in the left hind limb of B6D2F1 mice (8 animals/group) and, when the tumor mass was palpable (i.e., day 6 after tumor challenge), animals were treated with vehicle (PBS) or with the indicated doses of D16F7 mAb. Tumor growth was evaluated every other day. Results are the arithmetic mean of tumor volumes ± SD. Statistical analysis using the ANOVA and Bonferroni post-test method for multiple comparisons indicated that differences in tumor size between untreated and D16F7 mAb treated mice (both 10 and 20 mg/kg doses) were statistically significant starting from day 10 onward (*p* < 0.05). Differences in tumor size between mice treated with 10 and 20 mg/kg of D16F7 mAb were statistically significant starting from day 12 onward (*p* < 0.05). Data are representative of two independent experiments with similar results. (**C**) D16F7 mAb induces apoptosis in B16F10 melanoma cells *in vivo*. Hematoxylin and eosin staining of histological sections of melanoma samples obtained from control or mAb treated mice. Control mice show a tumor mass composed by vital cells with brisk mitotic activity (arrows in panel 60×) and very few area of necrosis in the tumor core. Treatment with D16F7 mAb (10 mg/kg) induces a degenerative aspect of the tumor mass with a high number of apoptotic cells (arrows). Histograms represent the mean percentage ± SD of tumor mass area occupied by apoptotic cancer cells or mean number ± SD of cells/10 high-power fields (HPF) displaying mitotic activity in control and treated tumors (10 and 20 mg/kg).

D16F7 serum levels and toxicity were evaluated in B6D2F1 mice to be used for antitumor *in vivo* studies. Antibody concentrations in mice serum were evaluated up to 7 days after the last of five doses administered intraperitoneally (i.p.) every other day. D16F7 steady state serum concentration in animals treated with 20 mg/kg was 3–4 higher than in mice treated with 10 mg/kg (Table 1). Values were largely above those required *in vitro* to inhibit endothelial, myelomonocytic and melanoma cell migration, melanoma vasculogenic mimicry and *in vivo* angiogenesis. Importantly, the observed circulating mAb levels did not cause toxic effects in treated animals. In fact, no significant changes in body weight were observed during mice treatment with D16F7, as compared with control animals treated with mAb solvent (PBS) (Figure 5A).

**Table 1:** Serum levels of D16F7 mAb in mice

Time after the last dose (days)^a^	D16F7 mAb concentration in mouse serum (μg/ml)^b^	
	10 mg/kg D16F7	20 mg/kg D16F7
1	496 ± 41	1185 ± 56
5	249 ± 28	889 ± 76
7	262 ± 33	888 ± 14

a Serum was prepared using blood samples obtained from D16F7 mAb treated (5 doses administered i.p. every other day) animals (4 mice per group) at the indicated times after injection of the last mAb dose.

b D16F7 levels in serum samples were determined by ELISA using 96-wells plates coated with VEGFR-1/Fc chimera.

D16F7 effect on *in vivo* tumor growth was evaluated by injecting intramuscularly (i.m.) B16F10 cells in syngeneic B6D2F1 mice. When tumor was palpable, animals were treated i.p. with 10 or 20 mg/kg of D16F7 on alternate days. A strong reduction in tumor volume was observed in animals treated with D16F7 as compared to control mice (Figure 5B, Table 2). In particular, on day 16 after tumor challenge melanoma growth inhibition after treatment with 10 and 20 mg/kg of D16F7 was 48.6% and 74.4%, respectively. D16F7 administration significantly increased the tumor growth quadrupling time, with tumor growth delay indexes of 1.54 (10 mg/kg) and 2.26 (20 mg/kg) (Table 2).

**Table 2:** Effect of D16F7 mAb treatment on tumor growth *in vivo*

Experimental group^a^	Tumor volume inhibition (%)^b^^,^^d^	Tumor growth quadrupling time (days)^c^^,^^d^	Tumor growth delay index^d^
Control mice	–	3.37 ± 0.16	–
10 mg/kg D16F7	48.6 ± 9.7	5.18 ± 0.27	1.54 ± 0.08
20 mg/kg D16F7	74.4 ± 15.0	7.63 ± 1.11	2.26 ± 0.33

a Mice were treated with vehicle (control mice) or 10 or 20 mg/kg D16F7 mAb on alternate days. D16F7 antitumor efficacy was assessed by the end-points described in the Materials and methods section.

b Tumor growth inhibition was calculated at the end of the experiment illustrated in Figure 5, on day 16 after injection of B16F10 melanoma cells (*n* = 5 animals/group).

c Tumor growth quadrupling time was calculated starting from day 6 (*n* = 8 animals/group).

d Results are mean values ± SD. Statistical analysis using the ANOVA and Bonferroni post-test method for multiple comparisons indicated that differences between tumor growth quadrupling times of treated (both 10 and 20 mg/kg) and control mice were statistically significant (*p* < 0.05). Differences in tumor volume inhibition, tumor growth quadrupling time and tumor growth delay index between 10 and 20 mg/kg treated mice were statistically significant (*p* < 0.05). Data shown are representative of two independent experiments with similar results.

Two weeks after treatment start three mice for each experimental group were sacrificed for tumor histological analysis. Hematoxylin and eosin staining showed that 10 and 20 mg/kg of D16F7 mAb had significant effect on melanoma cell viability, apoptotic cells being 37% of tumor mass *versus* 1% in control animals. Moreover, melanoma sections from D16F7 treated animals showed a drastically reduced mitotic activity compared with control sections (2–4 *versus* 33 mitosis/10 HPF for treated and control mice, respectively, Figure 5C).

**Figure 6:**
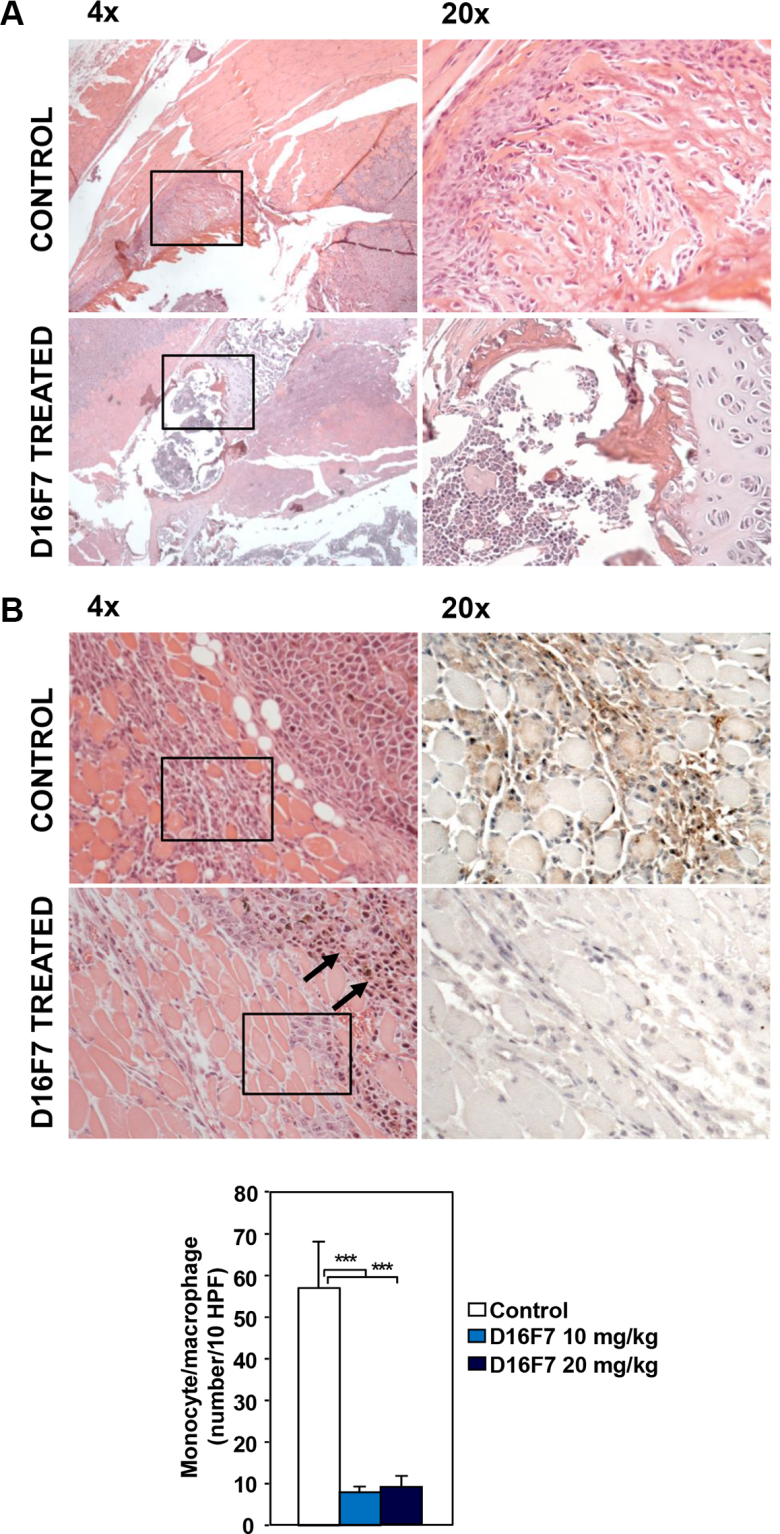
Treatment with D16F7 mAb inhibits bone infiltration by tumor cells and monocyte/macrophage infiltration at the tumor border (**A**) Bone infiltration by tumor cells. In control animals, hematoxylin and eosin staining of histological sections from mice left hind limb shows a marked infiltration of leg cortical bone by tumor cells (panel 4×). In D16F7 treated mice (10 mg/kg) bone infiltration is, instead, drastically reduced (panel 4×). Panels 20× display the amplification of the black-lined inset in the corresponding panels 4×. (**B**) Monocyte/macrophage infiltration at the tumor border. In the control group, hematoxylin and eosin staining shows that neoplastic cells at the muscular front are healthy and muscle fibers are infiltrated by a high number of inflammatory cells (panel 4×), mainly monocytic in origin, as shown by the F4/80 positive staining (panel 20×, amplification of the black-lined inset in panel 4×). Treatment with D16F7 mAb (10 mg/kg) induces a degenerative aspect of tumor cells (arrows in panel 4×; hematoxylin and eosin staining) at the infiltrative border. Muscle fibers show only scattered monocyte/macrophage cells (panel 4×), as indicated by the absence of positivity for F4/80 staining (panel 20×, amplification of the black-lined inset in panel 4×). Histogram represents the arithmetic mean number of monocytic cells at the infiltrative border/10 HPF ± SD in control and treated tumors (10 and 20 mg/kg).

Treatment with either D16F7 dose drastically reduced bone infiltration by melanoma cells as well (Figure 6A). Interestingly, a marked decrease in monocyte/macrophage infiltration at the tumor border was observed in melanoma sections obtained from D16F7 treated mice compared with control melanoma sections (Figure 6B).

**Figure 7:**
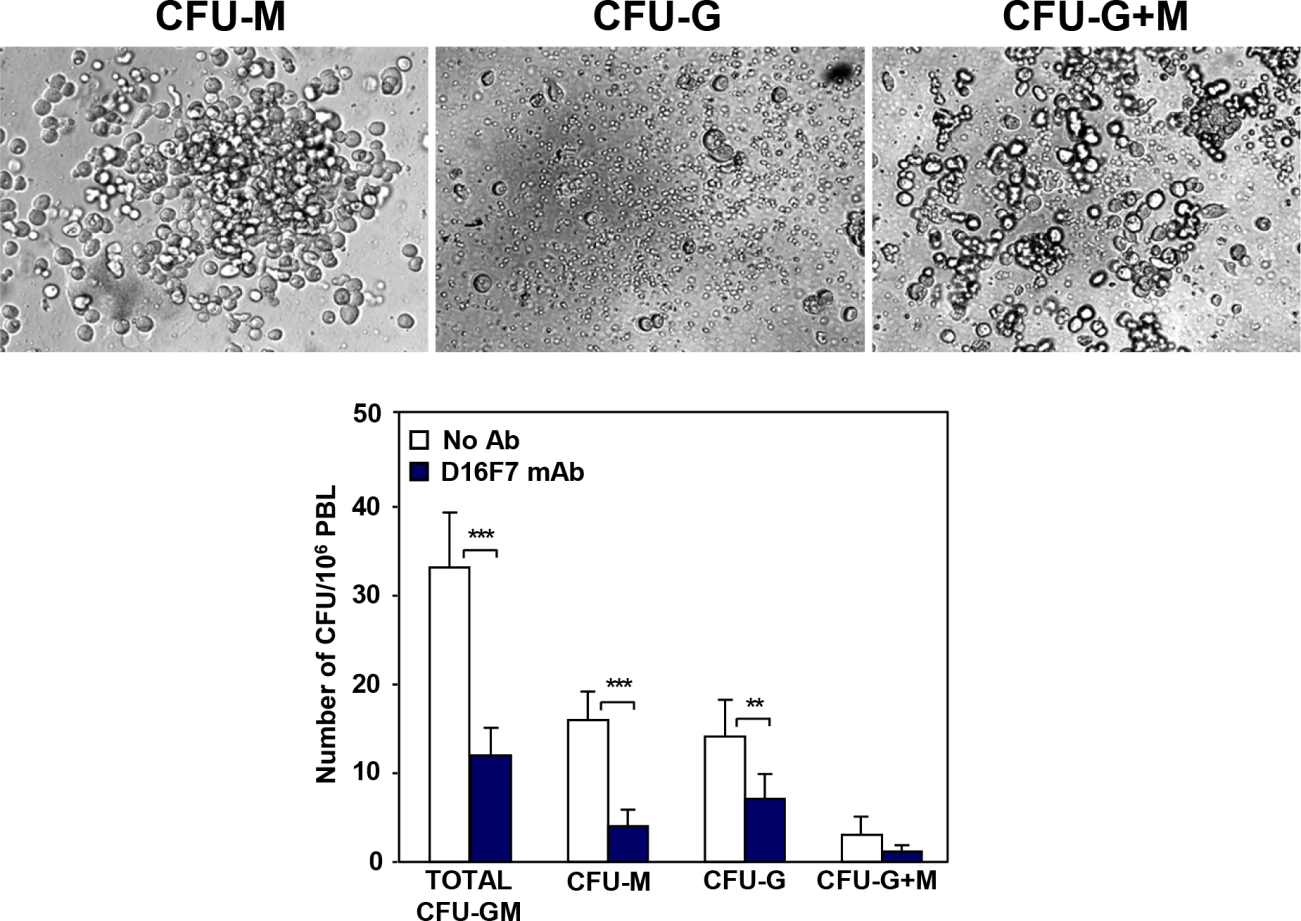
Treatment with D16F7 mAb decreases the number of myeloid progenitor cells in mice peripheral blood Leukocytes were isolated from pooled peripheral blood samples of control or D16F7 mAb treated B6D2F1 mice (20 mg/kg), mixed with MethoCult™ GF M3534 medium and seeded on 35 mm Petri dishes. Cells were allowed to grow in CO_2_ incubator at 37°C for 10 days. Colonies containing at least 30 granulocytes (CFU-G), monocytes/macrophages (CFU-M) or both cell types (CFU-G+M) were counted. The bar corresponding to total CFU-GM refers to the sum of CFU-G, CFU-M and CFU-G+M. The following morphological criteria (as shown in the photographs) were used: monocytic lineage cells are large cells with oval to round shape; granulocytic lineage cells are round, bright and much smaller but more uniform in size than macrophages. Histograms represent the arithmetic mean values of CFU number ± SD of a representative experiment out of three.

Since VEGFR-1 is known to be expressed in hematopoietic progenitor cells, D16F7 effect on the mobilization of these cells from the bone marrow was analyzed by evaluating their number in the peripheral blood of D16F7 treated *versus* control mice. Nucleated cells were isolated from the peripheral blood of the animals and mixed with MethoCult GF M3534 medium, which allows mouse hematopoietic progenitors of granulocytes and/or monocytes/macrophages to be detected and quantified using the colony formation assay (CFU-G, CFU-M and CFU-G+M). D16F7 treatment caused 64% reduction in myeloid progenitor cells mobilization compared with control mice: 75% reduction for monocyte/macrophage progenitors and 50% reduction for granulocytes progenitors (Figure 7).

No major differences were observed in the number of vascular structures surrounding or within the tumor mass, because angiogenesis induction was very limited in this melanoma model at the time the analysis was performed (i.e., 14 days after tumor challenge). Nevertheless, qualitative differences in vascular structures outside the tumor margin were observed between control and treated tumors. In fact, in control melanoma vessels were lined by normal endothelial cells, whereas in D16F7 treated melanoma they were lined by reactive endothelium with hobnail aspect and mitotic activity (Supplementary Figure S2).

Overall, treatment with D16F7 mAb had multiple effects on different parameters that resulted in reduced tumor growth and infiltrative potential without appreciable toxic effects.

D16F7 effect on tumor growth was compared with that of temozolomide (TMZ), an alkylating agent that has been extensively used for metastatic melanoma treatment [35]. D16F7 mAb (10 mg/kg) effect was similar to that obtained with temozolomide, with comparable values of tumor volume inhibition, quadrupling time and growth delay index (Table 3).

**Table 3:** Comparison of *in vivo* antitumor efficacy of D16F7 mAb and TMZ

Experimental group^a^	Tumor volume inhibition (%)^b^^,^^d^	Tumor growth quadrupling time (days)^c^^,^^d^	Tumor growth delay index^d^
Control mice	–	3.21 ± 0.68	–
10 mg/kg D16F7	49.7 ± 11.6	5.12 ± 1.40	1.59 ± 0.44
68 mg/kg TMZ	62.8 ± 9.8	5.36 ± 0.28	1.67 ± 0.09

a Mice (n = 6 animals/group) were treated with vehicle (control mice), 68 mg/kg TMZ for 5 consecutive days or 10 mg/kg D16F7 mAb on alternate days. The effect of D16F7 mAb or TMZ treatment on tumor growth was quantified by the parameters described in the Materials and methods section.

b Tumor growth inhibition was calculated at the end of the experiment on day 16 after injection of B16F10 cells.

c Tumor growth quadrupling time was calculated starting from day 10.

d Results are mean values ± SD. Statistical analysis using the ANOVA and Bonferroni post-test method for multiple comparisons indicated that differences between tumor growth quadrupling times of treated (either with D16F7 or TMZ) and control mice were statistically significant (*p* < 0.05).

Differences in tumor volume inhibition, tumor growth quadrupling time and tumor growth delay index between D16F7 and TMZ treated mice were not statistically significant.

## DISCUSSION

In the present study we described a novel mAb (i.e., D16F7) that recognizes VEGFR-1 and hampers its activation by VEGF-A or the VEGFR-1 specific ligand PlGF. D16F7 mAb is directed against a peptide whose amino acid sequence is comprised within the extracellular domain of the receptor and that inhibits receptor homodimerization and activity [29]. At variance with the other mAbs described in the literature that inhibit VEGFs/VEGFRs pathways, D16F7 does not prevent VEGFR-1 interaction with VEGF family members (e.g., VEGF-A and PlGF) but inhibits the cellular response that follows ligand binding to the receptor. This non-competitive mechanism of D16F7 action presents some advantages over mAbs that prevent receptor interaction with growth factors. In fact, due to its ability of not interfering with ligand binding to the extracellular domain of the receptors, D16F7 would not increase the amount of VEGF-A available to interact with and activate VEGFR-2. Moreover, D16F7 does not hamper sVEGFR-1 ability to act as decoy receptor for VEGF-A and PlGF, consisting in the sequestration of these angiogenic factors with consequent decrease in the amount of free ligands available to interact with membrane TKR. This is particularly important considering the previously suggested role of sVEGFR-1 in controlling tumor progression. In particular, lower expression of sVEGFR-1, specifically occurring in cutaneous metastases as compared to primary melanomas or lymph node metastases, may contribute to melanoma ability to invade the skin [30]. In addition, in other tumor models low sVEGFR-1/VEGF-A ratio has been related to higher aggressiveness [36]. Finally, since VEGFR-1 has 10-fold higher VEGF-A affinity compared to VEGFR-2, an anti-VEGFR-1 mAb that does not compete with growth factor binding to the receptor is likely to be effective at lower concentrations than a mAb that blocks the receptor through a competitive mechanism.

Soluble VEGFR-1 displays also a chemotactic effect on endothelial cells, indicating a role for this receptor isoform in angiogenesis [13]. Interestingly, pre-incubation of sVEGFR-1 with D16F7 down-modulated endothelial migration, suggesting that the mAb disrupts sVEGFR-1 interaction with the cells.

VEGFR-1 involvement in the induction of the angiogenic switch during pathological conditions, mobilization of precursor stem cells from the bone marrow and tumor growth and migration [4] supports the hypothesis of the therapeutic efficacy of targeting this receptor. Indeed, D16F7 mAb inhibited migration of endothelial cells triggered by VEGFR-1 ligands and neovessel formation in an *in vivo* matrigel plug assay, and caused morphological alterations of tumor-associated blood vessels. Additionally, D16F7 mAb inhibited the chemotactic response of VEGFR-1 expressing melanoma and myelomonocytic cells and exerted *in vivo* antitumor activity. Actually, the efficacy of 5 doses of 10 mg/kg of D16F7 was comparable to that of 5 doses of the anti-melanoma chemotherapeutic agent TMZ.

Besides being up-regulated in a variety of tumors, VEGFR-1 is expressed in monocyte/macrophages and involved in their recruitment to the tumor sites, where they secrete pro-angiogenic factors that further stimulate tumor growth and contribute to resistance to anti-VEGF-A therapies [37–39]. D16F7 mAb recognizes both human and murine VEGFR-1, likely due to a difference of two amino acids only between the peptide used as immunogen and the corresponding region of murine VEGFR-1. Therefore, we could analyze the effect of D16F7 treatment on tumor graft and host tumor microenvironment as well. Interestingly, tumor growth inhibition induced by D16F7 mAb was associated with reduced monocyte/macrophage cell infiltration at melanoma graft borders. Consistently, anti-VEGFR-1 mAb inhibited myeloid progenitor mobilization, as indicated by the marked decrease of CFU-M and CFU-G detected in the peripheral blood of tumor bearing mice.

Selective VEGFR-1 inhibition by D16F7 mAb might also potentiate the effects of VEGF-A targeting antiangiogenic therapies and counteract resistance development. Mechanisms of tumor resistance towards bevacizumab include increased VEGFR-1 expression (in tumor, endothelial cells and monocytes/macrophages) and signaling and/or up-regulation of the VEGFR-1 specific ligand PlGF [39–41]. Indeed, these mechanisms have been found to correlate with poor outcome of anti-VEGF-A therapy [39–41]. Moreover, we recently demonstrated that PlGF serum levels are significantly higher in melanoma patients than in healthy donors and treatment with VEGF-A blocking agents results in a further increase of PlGF levels [42]. Thus, the down-modulation of PlGF/VEGFR-1 pathway may delay or prevent resistance to anti-VEGF-A agents.

Resistance to anti-VEGF-A therapies may also be ascribed to blood vessels formation by mechanisms alternative to angiogenesis (e.g., intussusception, co-option, vasculogenic mimicry) [43]. Interestingly, VEGFR-1 has been shown to control melanoma vasculogenic mimicry [44, 45]. Accordingly, our results demonstrate a down-modulating effect of D16F7 mAb on capillary-like tubules formation by B16F10 melanoma cells *in vitro*, which might explain the high levels of apoptotic cancer cells observed upon D16F7 mAb treatment of tumor bearing mice *in vivo*. Indeed, the mAb did not induce apoptosis or inhibit tumor cell proliferation *in vitro*. Apoptosis and reduced proliferation of melanoma cells observed *in vivo* might also be favored by the low monocyte/macrophage infiltrate. Actually, tumor-associated macrophages, which are recruited by chemoattractants deriving from neoplastic and stroma cells, constitute the major fraction of tumor-infiltrating leukocytes. Besides cytokines, proteases, and chemokines that promote tumor angiogenesis they also express survival factors, which stimulate proliferation or inhibit apoptosis in tumor cells [46].

In spite of its involvement in tumor angiogenesis, VEGFR-1 does not play a relevant role in physiological angiogenesis in the adult. Therefore, antiangiogenic therapies selectively targeting this receptor are likely to be associated with lower systemic toxicity as compared to therapies targeting VEGF-A and/or VEGFR-2. Indeed, D16F7 mAb administration in a murine model was very well tolerated. On this basis, simultaneous VEGFR-1 targeting by D16F7 and VEGF-A blockade is likely to result in increased therapeutic efficacy without causing additive toxicity.

**Figure 8:**
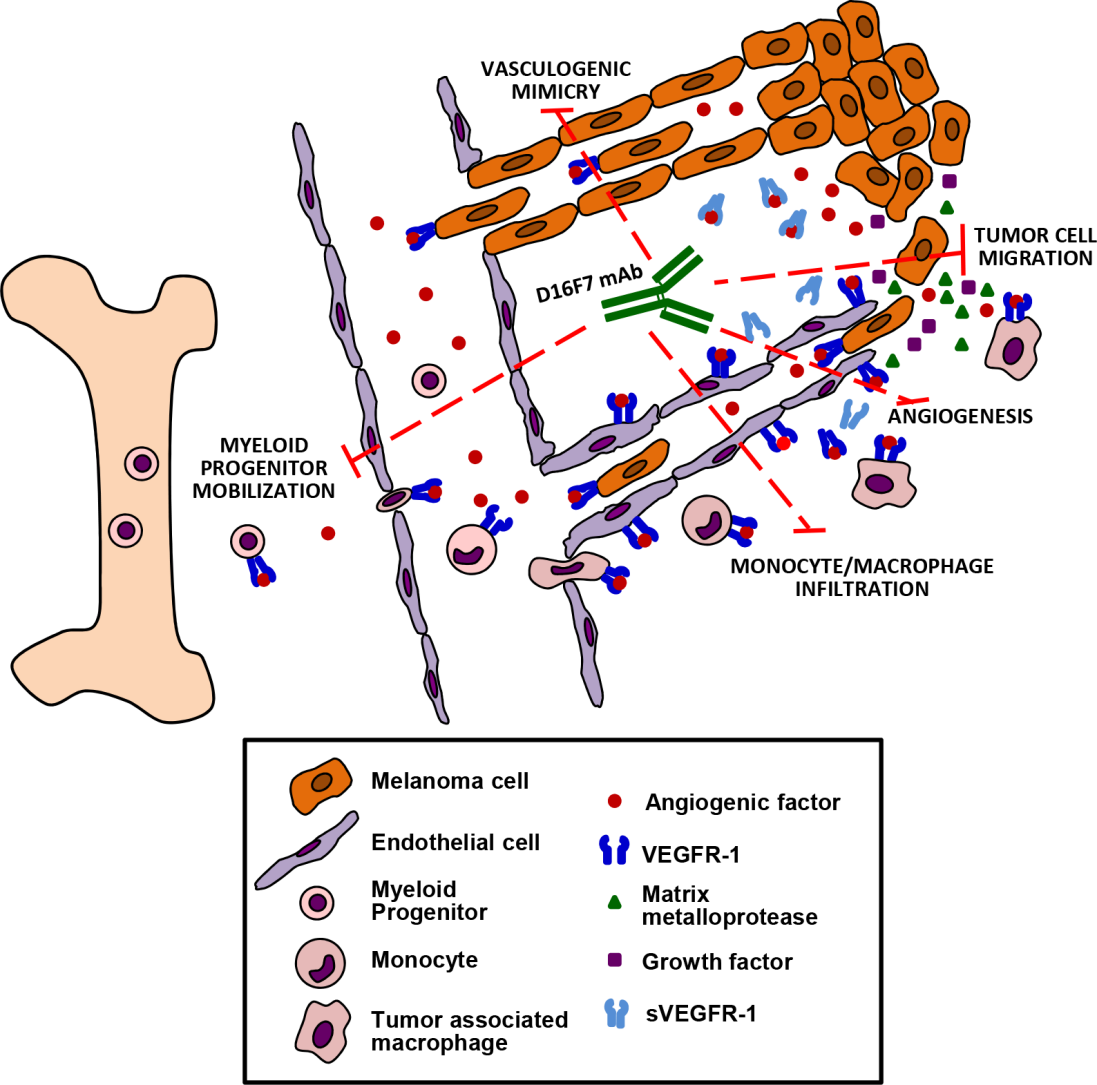
Schematic drawing summarizing the antitumor effects of D16F7 mAb Treatment with D16F7 exerts antitumor activity through several mechanisms of action: a) inhibition of angiogenesis after binding to endothelial cells, thus preventing chemotaxis induced by angiogenic factors and/or sVEGFR-1 released by tumor cells and/or tumor-associated macrophages; b) inhibition of myeloid hematopoietic progenitors mobilization and of tumor mass infiltration by monocytes/macrophages. The latter play an important role in tumor angiogenesis and invasiveness by secreting angiogenic factors, matrix metalloproteases and growth factors that promote tumor cell survival and metastases; c) inhibition of tumor growth and spreading of VEGFR-1 expressing tumor cells, by directly hampering chemotaxis and ability to form capillary-like tubes (i.e., vasculogenic mimicry), which allow nutrient supply and dissemination of metastatic cells.

In conclusion, we have generated a novel mAb specific for VEGFR-1 that displays potent *in vivo* antitumor activity, as a result of different effects: antiangiogenic activity, inhibition of tumor cell migration and vasculogenic mimicry, down-modulation of monocyte/macrophage recruitment to the tumor area and of myeloid progenitor mobilization from the bone marrow (Figure 8).

The obtained results strongly suggest that the D16F7 mAb might be used as a therapeutic agent in metastatic melanoma as well as other tumors or pathological conditions in which VEGFR-1 ligands, such as VEGF-A and PlGF, are involved.

## MATERIALS AND METHODS

### Reagents and cell cultures

Endothelial Basal Medium (EBM-2) and Endothelial Growth Factor Medium (EGM-2) were from Clonetics (BioWhittakerInc, Walkersville, MD); RPMI 1640 medium and other cell culture reagents were purchased from Lonza (Verviers, Belgium) and FBS from Euroclone (Milano, Italy). Fatty acid-free bovine serum albumin (BSA) was from Roche (Mannheim, Germany). Geneticin was from Invitrogen (Groningen, The Netherlands) and puromycin, heparin, PMA and other chemicals were from Sigma-Aldrich (St. Louis, MO). Recombinant VEGF-A and PlGF homodimers, polyclonal antibodies against VEGF-A or PlGF, and recombinant VEGFR-1/Fc, VEGFR-2/Fc, neuropilin-1/Fc (NRP-1/Fc) and platelet derived growth factor receptor β/Fc (PDGFR/Fc) chimeras were from R&D Systems (Abingdon, UK). Native human sVEGFR-1 was from ReliaTech (Braunschweig, Germany), recombinant human FGF-basic from Peprotech (Rocky Hill, NJ) and fibronectin from Sigma-Aldrich.

Human umbilical vein endothelial cells (HUVEC) were isolated from freshly delivered umbilical cords as previously described [13] and cultured in EGM-2. The immortalized human endothelial cell line HUV-ST [47] was maintained in culture in EGM-2 medium supplemented with 0.4 mg/ml geneticin and 5 μg/ml puromycin. The human melanoma cell line CR-Mel was established in the Laboratory of Molecular Oncology, IDI-IRCCS (Rome, Italy) [30] and cultured in 10% FBS/RPMI 1640.

The human promyelocitic HL-60 and murine melanoma B16F0 and B16F10 cell lines were obtained from ATCC and cultured in 10% FBS/RPMI 1640. The HL-60 cells were differentiated towards monocytic/macrophagic cells with 10 ng/ml PMA for 24 h.

### Generation of a mAb against VEGFR-1

Monoclonal antibodies were generated using as immunogen a peptide whose sequence corresponds to amino acids 149–161 from VEGFR-1 II IgG-like domain [29]. The peptide was utilized in its MAP (Multiple Antigenic Peptide) four branched form (obtained from PRIMM, Milan, Italy). Immunization of 5–6 week old female BALB/c mice (18–20 g weight; Charles River, Calco, Milan, Italy) and generation of anti-VEGFR-1 mAb producing hybridomas were performed according to standard protocols. Hybridoma D16F7 was selected based on its ability to produce a mAb (IgG1) that specifically recognizes VEGFR-1, as evaluated by immunoblot and ELISA analyses (Supplementary Figure S1).

A patent has been filed to protect the use of D16F7 mAb (patent number 102016000034933).

### Chemotaxis and ECM cell invasion

*In vitro* migration and invasion assays were performed using Boyden chambers equipped with 8 μm pore diameter polycarbonate filters (Nuclepore, Whatman Incorporated, Clifton, NJ) coated with 5 μg/ml gelatin (Sigma-Aldrich) or 20 μg of the commercial basement membrane matrix matrigel (BD Biosciences, Buccinasco, Italy), respectively, as previously described [29, 48]. In the experiments to test the effect of D16F7 mAb, melanoma cells were pre-incubated with the indicated amounts of D16F7 mAb or control antibody (mouse IgG1, R&D Systems) in a rotating wheel for 30 min at room temperature. Cells were then loaded in Boyden chambers and migration or invasion assays were performed in the presence of either antibody. Migrated cells, attached to the lower side of the filters, were stained with crystal violet counted in triplicated samples for a total of 12 high power (x200 magnification) microscopic fields.

### *In vivo* matrigel plug assay

D16F7 mAb ability to modulate neovascularization was evaluated by matrigel plug assay, using VEGF-A as stimulus, according to a previously described method [49]. Five hundred microliters of matrigel supplemented with VEGF-A (100 ng/ml) or with VEGF-A plus 10 μg/ ml D16F7 mAb or control antibody, were injected subcutaneously (s.c.) into the flank of 8 week old male C57BL/6 mice (24–25 g weight; Charles River Laboratories). After five days, mice were sacrificed, matrigel plugs removed and angiogenic response evaluated by macroscopic analysis and spectrophotometric measurement of hemoglobin content using the Drabkin's method [50].

### VEGF-A and PlGF binding to VEGFR-1

Quantification of VEGF-A and PlGF binding to VEGFR-1 was performed on 96-well Maxisorp Nunc immunoplates (Nunc, Roskilde, Denmark) coated with 10 μg/ml VEGFR-1/Fc chimera in PBS. After blocking of the plates with 3% BSA (w/v) in PBS (hereafter referred to as blocking buffer), 50 μl of PlGF or VEGF-A solution (50 ng/ml in blocking buffer) were added to selected wells and incubated for 2 h. Detection of the amount of VEGFR-1/Fc chimera bound cytokine was performed with biotinylated goat anti-VEGF-A or anti-PlGF antibodies and streptavidin-alkaline phosphatase-conjugate (1:10,000; Roche). After alkaline phosphatase reaction, optical density at 405 nm was measured in a Microplate reader 3550-UV (Bio-Rad, Hercules, CA). The specificity of VEGF-A or PlGF binding to VEGFR-1 was demonstrated by preincubating the growth factors with neutralizing antibodies, before adding them to the wells coated with VEGFR-1/Fc chimera, and background was determined in blocking buffer coated wells.

The effect of D16F7 mAb on VEGF-A or PlGF binding to VEGFR-1 was evaluated by pre-incubating selected sVEGFR-1 coated wells with 10 μg/ml of D16F7 or control mAb for 30 min at room temperature, before adding the growth factors to the plate.

### Analysis of VEGFR-1 homodimerization and heterodimerization

The interaction of VEGFR-1 with other VEGF-A receptors was evaluated in 96-well Maxisorp Nunc immunoplates coated with 10 μg/ml sVEGFR-1. After blocking with binding buffer (PBS containing 3% BSA, 1 mM CaCl_2_, 0.5 mM MgCl_2_) for 4 h at room temperature, 50 μl of chimeras, comprising the extracellular regions of different VEGFRs fused with the Fc region of human immunoglobulins (i.e., 2 μg/ml VEGFR-1/Fc, 4 μg/ml VEGFR-2/Fc and 1 μg/ml NRP-1/Fc), were added to selected wells and allowed to bind to VEGFR-1. Chimeras binding was detected using alkaline phosphatase-conjugated anti-human Fc antibody (1:5000; Sigma-Aldrich). After alkaline phosphatase reaction, optical density at 405 nm was measured in a Microplate reader 3550-UV. The effect of D16F7 mAb on VEGFR-1 interaction with chimeric proteins was analyzed by pre-incubating selected sVEGFR-1 coated wells with 5 μg/ml of D16F7 mAb for 30 min at room temperature before adding the chimeras to the plate.

### VEGFR-1 phosphorylation

VEGFR-1 auto-phosphorylation was analyzed using an ELISA kit (R&D Systems) and extracts from CR-Mel melanoma cells stimulated with VEGF-A or PlGF. Cells (1 × 10^6^/well) were seeded in 6-well plates, allowed to growth overnight in complete medium, starved for 6 h in serum-free medium (RPMI 1640 containing 0.1% BSA and 1 μg/ml heparin) and pre-incubated for 30 min at room temperature with D16F7 or control mAb (10 μg/ml) before stimulation for 10 min at 37°C with VEGF-A or PlGF (200 ng/ml). Cells were then washed twice with PBS and cell extracts prepared as indicated in the kit instructions using 250 μl of lysis buffer. Protein concentration in the samples was determined by Bradford assay, and 20 and 30 μg of proteins were used to determine the amount of total and phosphorylated VEGFR-1 in the extracts, respectively.

### RT-PCR analysis

cDNA preparation and PCR amplification to evaluate VEGFR-1 expression were performed as previously described [18], utilizing an annealing temperature of 58°C and the following primers: human VEGFR-1, forward primer 5′-CTCCTGAGTACTCTACTCCT-3′, reverse primer 5′-GAGTACAGGACCACCGAGTT-3′ (640 bp fragment); mouse VEGFR-1, forward primer 5′-GCAC CAAGAGCGATGTGTGG-3′, reverse primer 5′-ACACCA CGGAGTTGTAGTCT-3′ (749 bp fragment); mouse PlGF, forward primer 5′-ATGCTGGTCATGAAGCTGTTC-3′, reverse primer 5′-TCACGGGTGGGGTTCCTGA-3′ (477 bp fragment); GAPDH, forward primer 5′-TCCCATC ACCATCTTCCA-3′, reverse primer 5′-CATCACGCCA CAGTTTCC-3′ (380 bp fragment).

### Differentiation of B16F10 cells in tube-like structures

For tube-like structure analysis on matrigel, the matrix was thawed overnight at 4°C and diluted 1:3 in serum-free RPMI 1640 medium. Diluted matrigel (100 μl) was layered onto 24-well plates, which were incubated at 37°C for 30 min, until matrigel solidification. Cells were suspended in RPMI 1640 medium containing 0.1% BSA and 1 μg/ml heparin (4×10^4^ cells in 0.5 ml), dispensed onto solidified matrix and incubated at 37°C in 5% CO_2_ environment for 48 h. Plates were photographed using a Leica (Wetzlar, Germany) inverted microscope and Canon digital camera PowerShot G5 (Ōta, Japan). Tube-like structures were counted in ten different microscopic fields per group (×50 magnification).

### *In vivo* antitumor studies

To analyze the effect of D16F7 mAb on *in vivo* tumor growth, B16F10 cells (2.5 × 10^5^) were injected i.m. in the left hind limb of 6 week old male B6D2F1 inbred mice (24–26 g weight; Charles River). When tumor mass was palpable (i.e., 6 days after injection of melanoma cells), animals were treated i.p. with D16F7 mAb or TMZ, at the indicated doses and times, or the equivalent volume of vehicle alone.

Tumor growth was monitored every other day by measuring tumor mass three times a week in two dimensions by a caliper. Volumes were calculated according to the formula:

Tumor volume (cm^3^) = [length (mm) × width^2^ (mm^2^)]/2000

Antitumor efficacy of treatments was assessed by the following end-points:

- Percentage of tumor volume inhibition (TVI%) in treated *versus* control mice, calculated as:
  TVI % = 100 –[(TV treated/TV control) × 100]
- Tumor growth quadrupling time, calculated as the time required for tumor volume to increase 4-fold over initial volume at indicated times, using the formula:
  *t*_q_ = *t*_1_+ (*t*_2_−*t*_1_)log(V_q_/V_1_)/log(V_2_/V_1_)
  where: *t*_q_ is the interpolated quadrupling time; *t*_1_ and *t*_2_ are the lower and upper observation times bracketing the quadrupling tumor volume; *V*_q_ = 4*V*_0_, where *V*_0_ is the initial tumor volume; V_1_ and V_2_ are tumor volumes at the times *t*_1_ and *t*_2_, respectively;
- Tumor growth delay index, calculated as the mean treated/control tumor growth quadrupling time ratio.

Animals were euthanized, for ethical reasons, when tumor volume was 2.5–3 cm^3^.

Body weight (BW) was measured thrice-weekly and toxicity was evaluated on the basis of net BW reduction. The percentage of net BW variation between the first day of treatment and sacrifice day (or day of interest), was evaluated according to the following equation:

% net BW variation = [(net BW at observation day – net BW at first day of treatment)/net BW at first day of treatment] × 100

At sacrifice, tumors were excised, fixed in 10% buffered formalin solution (v/v), paraffin embedded and cut into 5 μm-thick slices for staining. A set of slides was stained with hematoxylin eosin for morphological study and mitosis counts. Additional slides were stained with anti-mouse PECAM/CD31 polyclonal antibody (M-20, Santa Cruz Biotechnology, Dallas, TX), to label blood vessels, or with rabbit monoclonal anti-mouse F4/80 antibody (SP115, Abcam, Cambridge, UK) to label monocyte/macrophage infiltrate. Reactions were revealed with 3,30-diaminobenzidine.

### Evaluation of hematopoietic progenitors in mice peripheral blood

Detection and quantification of mouse hematopoietic progenitors in peripheral blood was performed using nucleated cells isolated from the pooled peripheral blood of control or 20 mg/kg D16F7 mAb treated B6D2F1 mice (*n* = 4 for each experimental group) and mixed with MethoCult™ GF M3534 medium (StemCell Technologies, Vancouver, Canada). Briefly, 0.7–0.9 × 10^6^ cells were seeded in 35 mm Petri dishes (8 per group). Cells were allowed to grow for 10 days in CO_2_ incubator at 37°C and colonies containing at least 30 granulocytes (CFU-G), monocytes/macrophages (CFU-M) or both cell types (CFU-GM) were counted. Results were normalized by total number of initially plated cells.

### Evaluation of D16F7 mAb binding to VEGFR-1 and mouse serum levels

Quantification of D16F7 mAb binding to VEGFR-1 was performed on 96-well Maxisorp Nunc immunoplates coated with 10 μg/ml VEGFR-1/Fc chimera in PBS. After plate blockade with PBS containing 3% BSA, 50 μl of graded antibody concentrations in blocking solution were added to selected wells and incubated for 2 h. Amounts of D16F7 mAb bound to VEGFR-1/Fc chimera were detected using alkaline phosphatase-conjugated anti-mouse antibody (1:500; Sigma-Aldrich). After alkaline phosphatase reaction, optical density at 405 nm was measured in Microplate reader 3550-UV. Binding specificity and background were evaluated using PDGFRα/Fc chimera and BSA coated wells, respectively. The same assay was used to determine D16F7 mAb serum levels in treated mice, by serial dilutions of serum samples and including in the test a standard curve with graded concentrations of purified mAb (from 0.25 to 10 μg/ml).

### Animal care and ethics statement

All procedures involving mice and care were conducted in accordance with the ethical standards, according to the Declaration of Helsinki, in compliance with our institutional animal care guidelines and following national and international directives (D.L. March 4, 2014, no. 26; directive 2010/63/EU of the European parliament and council; Guide for the Care and Use of Laboratory Animals, United States National Research Council, 2011). Experimental protocols were approved by the Animal Care and Use Committee at the institutions involved in this study and by the Italian Ministry of Health.

### Statistical analysis

Results were expressed as arithmetic mean ± standard deviation (SD). Difference significance was tested by unpaired, two-tailed Student's *t-test*; *p value*s < 0.05 (*), < 0.01 (**) and < 0.001 (***) were considered to be significant. For statistical analysis of the results on *in vivo* tumor growth ANOVA followed by Bonferroni's pos*t-test* for multiple comparisons was used; a *p value* < 0.05 was considered significant.

## SUPPLEMENTARY MATERIALS AND FIGURES


