# Supplementary material for: Antitumor activity of a novel anti-vascular endothelial growth factor receptor-1 monoclonal antibody that does not interfere with ligand binding
Source: Oncotarget. 2016 Sep 19;7(45):72868–85. doi: 10.18632/oncotarget.12108 (PMC5341950; doi:10.18632/oncotarget.12108)
Supplement: Supplementary file 1 [file oncotarget-07-72868-s001.pdf]

Antitumor activity of a novel anti-vascular endothelial growth factor receptor-1 monoclonal antibody that does not interfere with ligand binding

Supplementary Materials

SUPPLEMENTARY MATERIALS AND METHODS

Western blot analysis

Samples (VEGFR-1/Fc or VEGFR-2/Fc chimeras) were run in 10% SDS-polyacrylamide gels and proteins transferred to supported nitrocellulose membranes for Western blotting analysis using the following primary antibodies at 1 µg/ml: anti-VEGFR-1 (AF321, R&D) and anti-VEGFR-2 (AF357, R&D) polyclonal Abs or D16F7 mAb. Anti-mouse or anti-rabbit Ig/Horseradish peroxidase secondary antibodies and ECL Western blotting detection reagents (GE Healthcare, Milan, Italy), were used to identify the proteins of interest.

Cell proliferation assay

Cell proliferation was evaluated in 96-well plates using the tetrazolium compound MTS [3-(4,5-dimethylthiazol-2-yl)-5-(3-carboxymethoxyphenyl) 2-(4-sulphophenyl)-2H-tetrazolium, inner salt] from Promega (Madison, WI), as previously described [47]. Graded amounts of cells suspended in complete medium containing 5 µg/ml of D16F7 or control antibody or without antibodies were dispensed into flat-bottom 96-well plates and grown at 37°C in a 5% CO_2 humidified atmosphere. Six replica wells were used for every condition. After 3-5 days, 20 µl of MTS solution were added to each well and cells were incubated at 37°C for 2 h. Absorbance was read at 490 nm (reference wavelength 655 nm) using a 3550-UV microplate reader.

Apoptosis analysis by flow cytometry

For apoptosis analysis after treatment of B16F10 cells with D16F7 mAb, cells were seeded in 6-well plates (2.5×10^5 cell/well) and, after 24 h, left untreated or treated with 5 µg/ml of D16F7 or control mouse IgG mAb at 37°C for 48 and 72 h. Cells were then harvested, washed with PBS and fixed in 70% ethanol at -20°C for 18 h. After centrifugation, cells were resuspended in 1 ml of hypotonic solution containing 50 µg/ml propidium iodide (Sigma-Aldrich), 0.1% sodium citrate, 0.1% Triton-X, and 10 µg/ml RNase (Roche), and incubated at 37°C in the dark for 15 min. The propidium iodide fluorescence was measured on a linear scale using a FACSscan flow cytometer (Becton Dickinson, Franklin Lakes, NJ) to detect the hypodiploid peak characteristic of apoptotic cells.

Cell adhesion to ECM components

Cell adhesion was tested on 96-well plates previously coated with commercial basement membrane matrix matrigel (20 µg/ml; BD Biosciences), collagen I (10 µg/ml, Roche), fibronectin (10 µg/ml, Sigma-Aldrich), gelatin (5 µg/ml, Sigma-Aldrich) and blocked with 3% BSA in PBS. Cells were seeded into wells (4×10^4 cells/well) and incubated at 37°C for 40 min. Non-adherent cells were washed out and attached cells fixed in ethanol for 5 min and stained with 0.5% crystal violet. Adhesion efficiency was determined by quantitative dye extraction with 10% acetic acid and spectrophotometric measurement of absorbance at 595 nm in a Microplate reader 3550-UV.

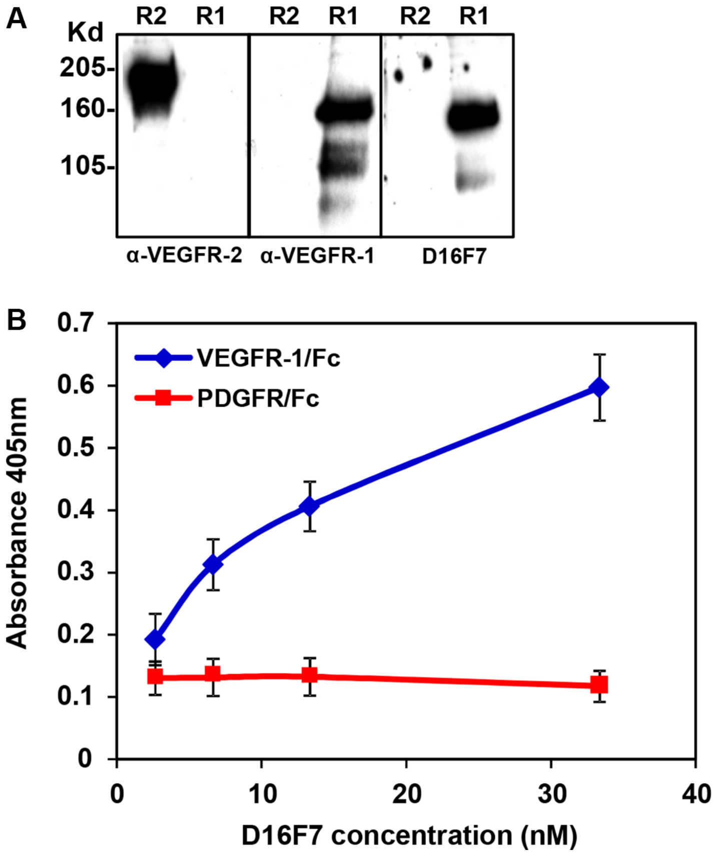

Supplementary Figure S1: Characterization of anti-VEGFR-1 D17F7 mAb. (**A**) D16F7 mAb specifically recognizes VEGFR-1 in immunoblot analysis. VEGFR-1/Fc (R1) or VEGFR-2/Fc (R2) chimeras were loaded on 10% polyacrylamide gels and anti-VEGFR-1 or anti-VEGFR-2 polyclonal Abs or D16F7 mAb were used for immunodetection. (**B**) D16F7 mAb specifically binds VEGFR-1 in a concentration-dependent manner. Titration of purified D16F7 mAb was performed by ELISA, using plates coated with VEGFR-1/Fc or PDGFRα/Fc chimeras (negative control). D16F7 mAb binding was assessed using alkaline phosphatase-conjugated anti-mouse antibody. Results are expressed as arithmetic mean ± SD of absorbance at 405 nm after subtraction of background absorbance measured in BSA coated wells. The experiment shown is representative of three independent determinations with similar results.

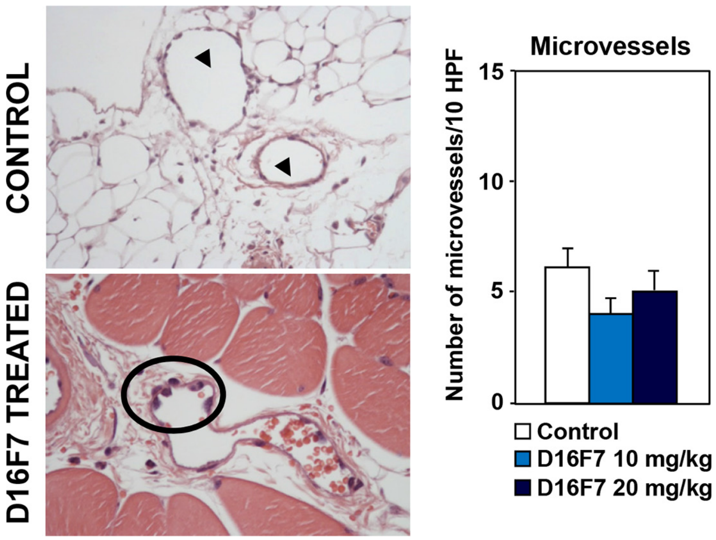

Supplementary Figure S2: Characterization of vasculature structures in melanoma specimens obtained from control or D16F7 mAb treated mice. In control mice, vascular structures are lined by normal endothelial cells in the tissue surrounding the tumor mass (arrow heads). In treated mice (10 mg/kg) extra-tumoral vessels are lined by reactive endothelium (circle). Original magnification, 4x. Histograms represent the number of microvessels/10 HPF ± SD.
